# Supplementary material for: Timing the escape of a photoexcited electron from a molecular cage
Source: Nat Commun. 2025 May 31;16:5062. doi: 10.1038/s41467-025-60260-z (PMC12126515; doi:10.1038/s41467-025-60260-z)
Supplement: Supplementary file 1 — Supplementary information [file 41467_2025_60260_MOESM1_ESM.pdf]

Timing the Escape of a Photoexcited Electron from a Molecular Cage: Suppl. Info.

Connor Fields[1], Aleksandra Foerster[2], Sadegh Ghaderzadeh[2], Ilya Popov[2], Bang Huynh[2], Filipe Junqueira[1], Tyler James[1], Sofia Alonso Perez[1], David A Duncan[2,3], Tien-Lin Lee[3], Yitao Wang[1], Sally Bloodworth[4], Gabriela Hoffman[4], Mark Walkey[4], Richard J Whitby[4], Malcolm H Levitt[4], Brian Kiraly[1], James N O'Shea[1], Elena Besley[2*], Philip Moriarty[1*]

[1]School of Physics & Astronomy, University of Nottingham, University Park, Nottingham, NG7 2RD, UK.
[2]School of Chemistry, University of Nottingham,University Park, Nottingham, NG7 2RD, UK.
[3]Diamond Light Source, Harwell Science & Innovation Campus, Didcot, OX11 0QX, UK.
[4]School of Chemistry, University of Southampton, Southampton, SO17 1BJ, UK.

*Corresponding author(s). E-mail(s): elena.besley@nottingham.ac.uk; philip.moriarty@nottingham.ac.uk;

Abstract

This supplementary information file comprises additional data and analysis for the following experimental and theoretical aspects of the *Timing the Escape of a Caged Electron* article: **(i)** Valence band photoemission for bulk Ar@C_{60}; **(ii)** Comparison of Ar $2p$ core-level photoemission and Ar $2p \to 4s$ X-ray absorption spectra; **(iii)** Extended discussion on fitting the Auger-Meitner decay spectra; **(iv)** Comparison of photoemission lineshapes for bulk and monolayer Ar@C_{60} samples; Doniach-Sunjic asymmetry; **(v)** Raw on-resonance decay spectra for multilayer and monolayer Ar@C_{60} coverage; **(vi)** Population analysis: Ar and C contributions to excited state; **(vii)** Orbital symmetry decomposition; **(viii)** Comparison with time-dependent DFT; **(ix)** Relativistic considerations; **(x)** Z+1 approximation and ground state K@C_{60}; and **(xi)** Ar-Ag(111) separation: Additional DFT calculations.

Supplementary Note 1. Valence band photoemission for bulk Ar@C_{60} film

The valence band spectrum for a thick film of Ar@C_{60} is shown in Supp. Fig.1. As observed previously by Morscher *et al.*[1], the valence band spectra of empty C_{60} and Ar@C_{60} are indistinguishable down to a binding energy of $\sim$ 10 eV below the Fermi level.

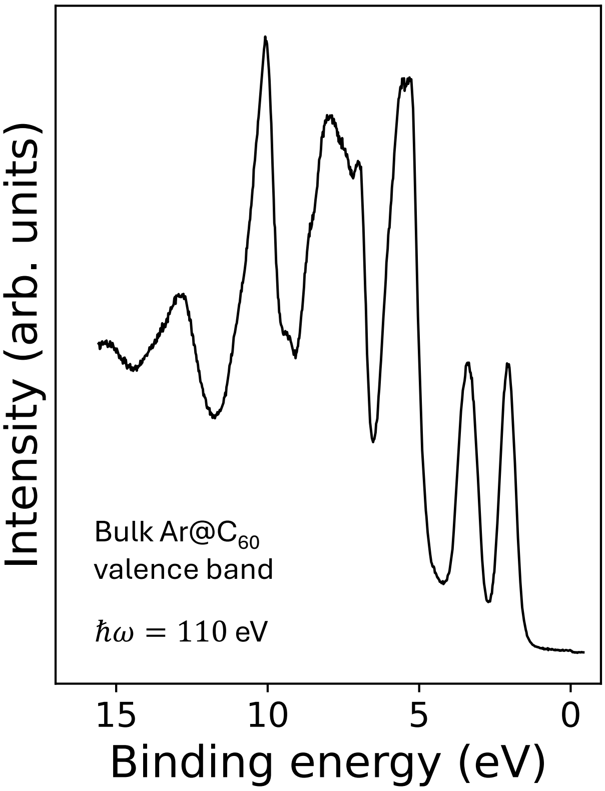

Supp. Fig. 1. Valence band spectrum for a bulk film of Ar@C_{60} acquired with a photon energy of 110 eV. 0 eV binding energy represents the Fermi level position. Source data provided as a Source Data file.

Supplementary Note 2. Ionic vs excitonic: Ar 2p XPS-XAS comparison

Supp. Fig. 2 shows the Ar $2p_{3/2} \rightarrow 4s$ X-ray absorption resonance in relation to the Ar 2p ($L_{2,3}$) spectrum acquired over a wider photon energy range. Wurth *et al.*[2] have previously identified the origin of the higher lying resonances. In the inset to Supp. Fig. 2 we show a comparison of the Ar 2p core-level and $2p_{3/2} \rightarrow 4s$ X-ray absorption spectra, plotted on the same energy scale. (The binding energy (BE) of the core-level spectrum is referenced to the Fermi level.) There is a difference of 2.7 ± 0.1 eV between the Ar $2p_{3/2}$ core level BE and the peak energy of the $2p \rightarrow 4s$ X-ray absorption spectrum, arising from the ionic (photoemission) vs neutral (excitonic, X-ray absorption) character of the final state of each process.

As highlighted by Martensson *et al.*[3] and Sandell *et al.*[4, 5] in the context of argon adsorption on graphite, the observation that the Ar $2p_{3/2}$ binding energy is significantly *lower* than that of the X-ray absorption peak is already a clear indication that the ionic state is considerably more energetically favourable than the excitonic, charge neutral state – this is ultimately the driving force for transfer of the 4s electron to the environment. We note that the difference of 2.7 ± 0.1 eV is rather higher than the value of 2.1 eV observed for argon on graphite[4], but identical to the 2.72 eV

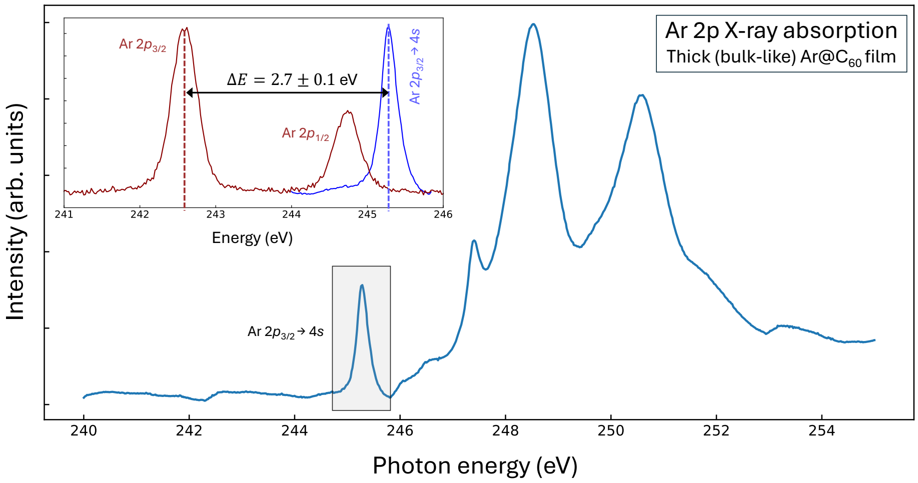

Supp. Fig. 2. Ar 2p photoemission and X-ray absorption spectra for the bulk Ar@C_{60} sample. Wide-energy-range X-ray absorption spectrum for energies close to the Ar 2p (i.e. $L_{2,3}$) absorption edges, measured by recording the sample drain current as a function of photon energy – a total electron yield measurement. Throughout the work described in the main paper and this supplement, we have focussed on absorption across the Ar $2p_{3/2} \rightarrow 4s$ resonance highlighted by the gray rectangle. See Wurth *et al.*[2] for a description of the origin of the other, higher lying resonances in the context of condensed "bare" argon multilayers. **Inset:** Ar 2p core-level photoemission spectrum (in red) plotted on same energy axis as the Ar $2p \rightarrow 4s$ X-ray absorption spectrum (in blue). The energy of the core-level photoemission spectrum is referenced to the Fermi level and the $2p_{3/2}$-related spectra have been normalised to equivalent heights for ease of comparison. Source data provided as Source Data files.

value reported by Lizzit *et al.*[12] for argon on a weakly coupled graphene monolayer on a ruthenium substrate. (Oxygen intercalation was used to reduce the coupling to the metal surface.)

As the photoemission binding energy, E_{XPS}, is referenced to the Fermi level, E_{F}, the difference between this and the X-ray absorption energy, i.e. $\Delta E = E_{\mathrm{XAS}} - E_{\mathrm{XPS}}$ is generally taken to represent the energy of the excitonic state with respect to E_{F}. This would in turn place the Ar 4s state at 4.90 ± 0.15 eV above the HOMO level (see Supp. Fig. 1 for a valence band spectrum.) Lizzit *et al.*[12] probe the alignment of the Ar 4s level with the empty density of states of graphene in this way; we adopt a similar approach in the main paper to ascertain the extent to which the core-excited Ar 4s state might align energetically with the s-SAMO (superatomic orbital) level. We note, however, that while the agreement with the 4.9 eV separation of the s-SAMO level from the HOMO in the calculations of Zhao *et al.*[8] is certainly noteworthy, the alignment of energy levels calculated from our experimental data in this way ignores the possible influence of the Ar 2p core hole on the HOMO and s-SAMO states of the fullerene cage.

Supplementary Note 3. Fitting Auger-Meitner decay spectra

Although the positions and relative intensities of the primary, easily identifiable spectator and normal Auger-Meitner features (at binding energies of ∼ 26.8 eV, 28.5 eV, 30.8 eV (on resonance), 33.1 eV (on resonance), 31.7 eV, and 32.1 eV) in the map and spectra shown in Fig. 2 of the main paper are in line with many previous studies (including [9–15]), there is one significant difference. We found a particular issue with fitting the peaks between binding energies of ∼ 29 eV and 30 eV – it was not possible to robustly track the normal Auger- Meitner feature that appears at an on-resonance binding energy of ∼ 30 eV throughout the entire 1 eV width of the resonance region while keeping the intensity ratio for all normal Auger-Meitner peaks constant, not least because this feature overlaps heavily with the spectator contribution. This necessarily leads to a higher level of uncertainty in the division of spectral intensity.

Supp. Fig. 3 shows a comparison of fits of the decay spectra either side of the resonance condition ($\hbar\omega = 245.3$ eV). Our strategy in fitting all decay spectra was to fully constrain all peak positions and peak widths, i.e. to allow no freedom in those parameters as a function of photon energy. In other words, the fitted binding energy of the spectator peaks across the resonance does not vary, and the normal Auger-Meitner peaks rigidly change their binding energy in line with the variation in photon energy (i.e. a change of 20 meV from spectrum to spectrum). In addition, the relative intensities of the spectator peaks (including the shake-up components) are fully constrained. However, in order to provide satisfactory fits, we allowed variation in the relative intensities of the Auger-Meitner peaks, particularly in the 29 eV to 30 eV range.

Attempts to account for this spectral intensity by fitting an additional, shifted normal Auger-Meitner contribution arising from a small residual contribution from the first Ar@C_{60} monolayer directly bonded to the Ag(111) substrate (see Supp. Notes 4 and 5 below) were not successful. Our motivation in attempting to introduce this contribution was informed by our sample preparation strategy: depositing the argon endofullerene onto a cold substrate significantly limits molecular mobility and it is very unlikely that the film grows in a simple layer-by-layer mode. Indeed, the slow rate of decay of the Ag $3d$ and Ag(111) valence band photoemission intensity as a function of deposition time would point to significant islanding, with regions of the first chemisorbed monolayer remaining exposed up to high molecular coverages. (There is an appreciable Ehrlich–Schwoebel barrier ($\sim$ 120 meV) for C_{60} diffusion[16].)

However, a number of observations led us to discount the possibility of a residual contribution to the multilayer/bulk spectra arising from the monolayer: (i) as discussed in the main paper and Supp. Note 5 below, there is a significant, 1.25 eV, shift in the binding energies of the normal Auger-Meitner peaks for the endofullerene monolayer as compared to the bulk film. The normal Auger-Meitner intensity in the 29 − 30 eV binding energy range is not in line with this shift; (ii) as discussed in the following section, there is a very clear binding energy difference in the C $1s$ and Ar $2p$ core-level emission for the monolayer vs the bulk film that would produce a strong shoulder in the spectra should the monolayer make more than a negligible contribution. We did not observe a shoulder of this type in the multilayer specta; and (iii) the monolayer Auger-Meitner emission has a slightly higher on-resonance energy than that for the bulk film (245.4 eV vs 245.3 eV), out of line with the behaviour of the spectral intensity in the 29-30 eV binding energy (on resonance) range.

There is one final key point on the subject of fitting the decay spectra that we would like to raise. Given the large number of constrained parameters, uncertainties that are returned via the standard approach, i.e. diagonalisation of the covariance matrix produced via the non-linear least squares fitting routine (which we implemented using the Python LMFIT package), are often significantly underestimated (and, moreover, assume normally distributed errors.) In order to provide a more robust estimate of the uncertainties, we therefore employed a Monte Carlo/bootstrapping approach to explore the fitness landscape. Details of this approach are given in the caption for Supp. Fig. 4. For decay spectra close to resonance (i.e. those excited with photon energies within ± 100 meV of the on-resonance value of 245.3 eV), we estimate that the value of τ_D can be determined to an uncertainty of approximately 5%. Outside of this photon energy range, the signal-to-noise ratio is appreciably larger and the estimated uncertainties consequently significantly larger (up to 10%).

Supp. Fig.4 shows the variation in τ_D as a function of photon energy around the resonance condition. There is a monotonic decrease in the delocalization time across the resonance, which we have fitted with a simple linear dependence. The magnitude of the uncertainties (whose estimation is described in the caption for Supp. Fig. 4) does not justify a more sophisticated/higher order fitting function.

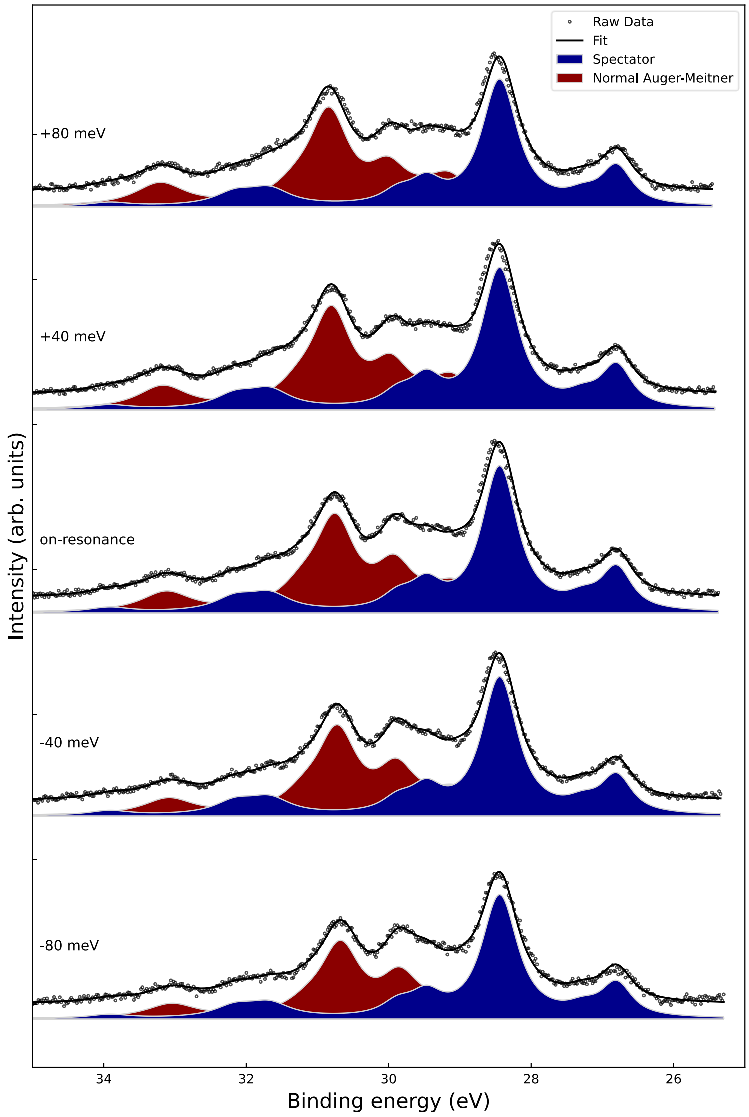

Supp. Fig. 3. Fitted decay spectra. A set of decay spectra, with accompanying fits, acquired with photon energies either side of the on-resonance condition ($\hbar\omega = 245.3$ eV). Source data available as Source Data files.

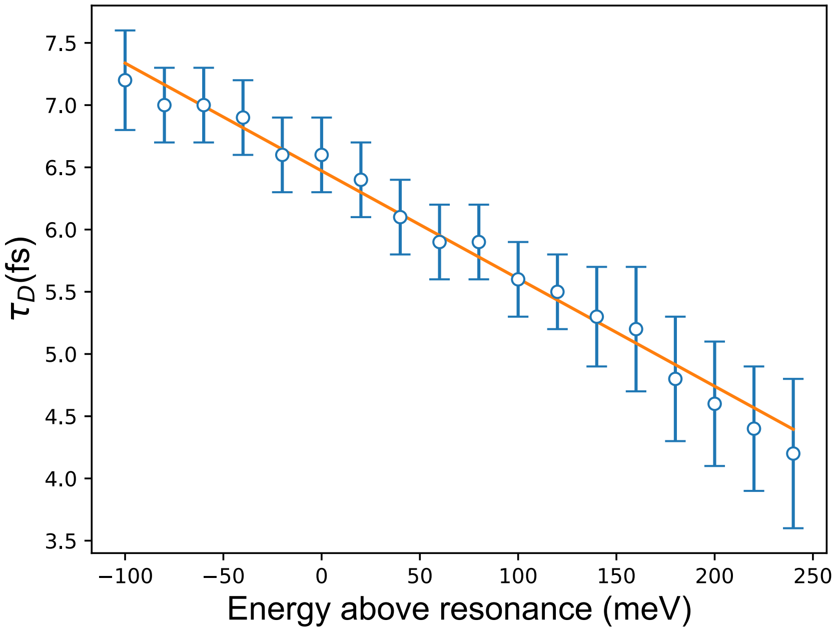

Supp. Fig. 4. Variation of delocalisation time with photon energy. There is a monotonic decrease in τ_D with increasing photon energy in this range. We have fitted a linear dependence. Other functional forms could in principle also be used to fit the data, including an exponential decay in line with the dependence of tunnelling rate on effective barrier height, but are difficult to justify due to the magnitude of the uncertainties. A Monte Carlo sampling/bootstrapping approach was used to calculate the error bars. We first calculated the best fit function for a given spectrum using the non-linear least squares fitting methodology underpinning the Python `lmfit` package. The standard deviation of the residuals spectrum was then used to provide a measure of noise to be added to the best fit function in order to generate synthetic spectra. In addition, and of key importance, the intensity of each of the traditional Auger-Meitner components was allowed to vary during the fit; our motivation here was to account as best as we could for uncertainties in the overall intensity (and distribution of component intensities) of the normal Auger-Meitner contribution to the decay spectra. However, the relative intensities of the spectator Auger-Meitner components were held rigidly fixed throughout this bootstrapping process. Each of the synthetic spectra was then also fitted using `lmfit`, and a value for τ_D determined. One hundred synthetic spectra (for each photon energy) were generated. The mean (open circles) and standard deviation (error bars) of the 100 values of τ_D determined in this way are plotted above. Source data provided as a Source Data file.

Although better fits – i.e., lower χ^2 values and, nominally, lower uncertainties – could be obtained by allowing small (i.e. few percent) variations in all peak positions, widths, and intensity ratios, we rejected this fitting strategy. Given the exceptionally large parameter space, we preferred instead, and as noted above, to heavily constrain the fits on the basis of the relevant physics/physical chemistry of the system. (As von Neumann famously said, "*With four parameters I can fit an elephant, and with five I can make him wiggle his trunk.*"[17].)

Supplementary Note 4. Comparison of photoemission spectra for bulk and monolayer Ar@C_{60}

There is a substantial shift in both C 1*s* and Ar 2*p* core-level binding energy (CLBE) for Ar@C_{60} molecules that are bound in the first monolayer to the Ag(111) surface as compared to the CLBE for endofullerenes in higher (physisorbed) layers. This BE shift has also previously been observed for empty C_{60} on Ag(111)[20] (see below for a detailed comparison). The main panel in Supp. Fig. 5 highlights the BE shift (but obscures the difference in C1*s* lineshape for monolayer vs bulk Ar@C_{60} samples – we'll return to this soon.) Argon 2*p* photoemission spectra (inset to right in Supp. Fig. 5) show the same 400 meV binding energy difference for monolayer versus bulk Ar@C_{60} samples as observed for the C 1*s* data.

A bulk film of Ar@C_{60}, sufficiently thick so that minimal Ag 3*d* emission from the Ag(111) susbtrate is observed, yields the C 1*s* spectrum shown in the upper left inset to Supp. Fig. 5. This, and the associated shake-up spectrum (also shown in Supp. Fig. 5), are identical to the corresponding spectra for empty C_{60}, both in terms of line shape and binding energy.

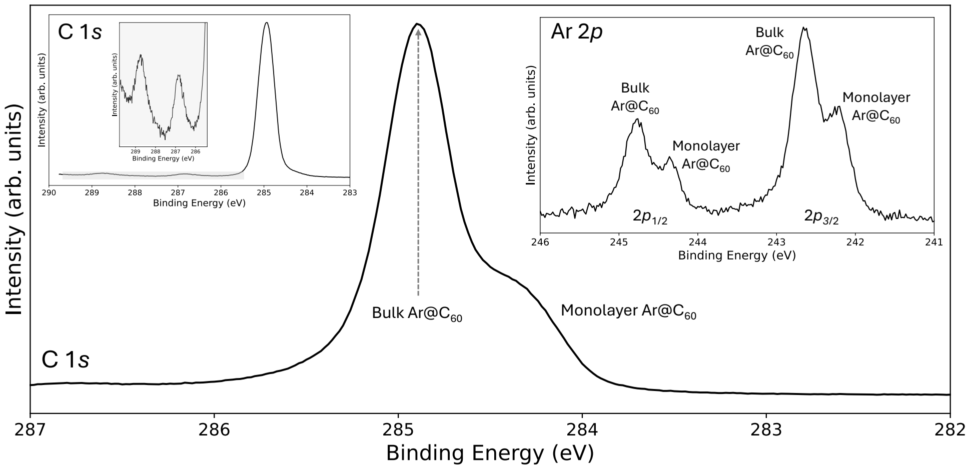

Supp. Fig. 5. Comparison of photoemission spectra for monolayer and multilayer coverages. The main figure is a C 1*s* photoemission spectrum (acquired with a photon energy of 400 eV) of a multilayer coverage of Ar@C_{60} on Ag(111) that clearly shows the significant (400 meV) difference in core-level binding energy associated with endofullerene molecules in the first monolayer and in higher layers. **Inset to left:** C 1*s* spectrum for a bulk-like Ar@C_{60} film sufficiently thick to "quench" Ag 3*d* photoemission intensity from the Ag(111) substrate on which it was grown. The shake-up features are magnified in the inset. This spectrum is indistinguishable from that of empty C_{60}. **Inset to right:** Ar 2*p* photoemission spectrum ($\hbar\omega$ = 400 eV) for the same multilayer sample as shown in the main figure. Again, there is a 400 meV shift towards lower binding energy for photoemission from molecules in the first monolayer, as compared to those in higher layers. Source data available as Source Data files.

Lineshape analysis: Doniach-Sunjic asymmetry

Although the position of the argon atom inside the endofullerene is unaffected by the significant level of charge transfer from the Ag(111) surface into the cage at the molecule-surface interface (see main paper), the Ar 2*p* photoemission spectrum for a monolayer coverage of Ar@C_{60} on Ag(111) nonetheless shows the signature asymmetry, i.e. the Doniach-Sunjic (D-S) lineshape[18], arising from excitation of the conduction electron density of the underlying metal (Supp. Fig. 6). As also observed by both Pedio *et al.*[20] and Gibson *et al.*[21] for 1ML of empty C_{60} on Ag(111), the C 1*s* core-level spectrum is similarly highly asymmetric. (See below.) In other words, while the encapsulated argon atom is unaffected by endofullerene adsorption from the perspective of its intracage position, it is nonetheless highly sensitive to the surrounding electrostatic and electrodynamic environment[22].

Notably, the asymmetry parameters for the D-S lineshapes are very similar – 0.243 ± 0.007 (C 1*s*) and 0.28 ± 0.01 (Ar 2*p*), indicative of a common origin. (The fitting is complicated somewhat by the presence of a small amount of endofullerene above the first monolayer due to the sample preparation process, where a small "overshoot" in coverage is difficult to avoid. However, the relatively large BE shift of 0.4 (±0.1) eV between the Ar 2*p* core-level signal for the first endofullerene layer and for subsequent layers facilitates easy identification of this contribution to the photoemission spectra. The endofullerene molecules above the first layer also make a characteristic contribution to the low energy electron diffraction (LEED) pattern, forming a weak

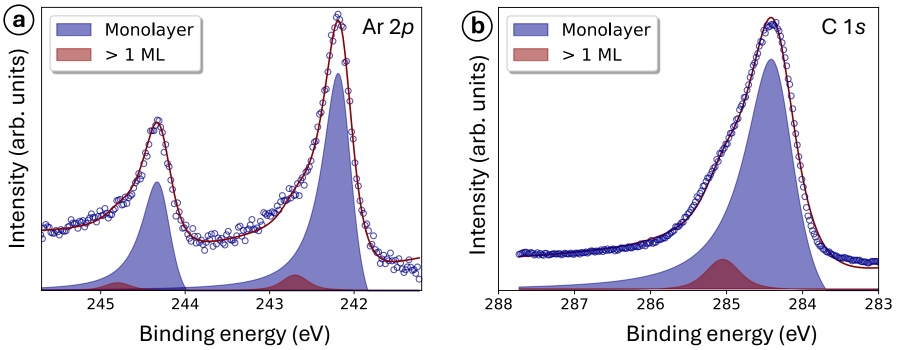

Supp. Fig. 6. Doniach-Sunjic asymmetry in monolayer photoemission lineshapes. The **(a)** Ar 2*p* and **(b)** C 1*s* photoemission lineshapes ($\hbar\omega$ = 400 eV) for the Ar@C_{60} monolayer show a pronounced asymmetry to higher binding energy that arises from the response of the metallic conduction electron gas to the creation of the core hole[18, 19]. We have fitted the asymmetric lineshape in each case to a Doniach-Sunjic function[18], but also have to account for an additional asymmetry due to a small amount of additional adsorbed endofullerene above the first monolayer (shaded in purple in the fits above). Each peak compromises two components: one for the monolayer (fitted with an asymmetric DS profile), and a second, much lower intensity, contribution at higher binding energy arising from physisorbed endofullerene on top of the chemisorbed monolayer. The contributions from the physiorbed molecules are each fitted with a symmetric Voigt lineshape. The asymmetry parameters, α, for the C 1*s* and Ar 2*p* spectra, 0.243 ± 0.007 and 0.28 ± 0.02, respectively, are very similar. (The error bars are determined from the covariance matrix of the fit.) Source data available as Source Data files.

multi-domain pattern that is distinct from the $(2\sqrt{3} \times 2\sqrt{3})$R30° superlattice.)

In order to better compare our Ar@C_{60} photoemission data with previously published (and extensive) work on the empty C_{60}-on-Ag(111) system[20], we have artificially broadened (via numerical convolution) our C 1s spectrum for the close-to-1-ML Ar@C_{60} sample with a Gaussian having a full width at half maximum of 0.4 eV. This is to match the experimental resolution reported by Pedio *et al.*[20] for their C 1s X-ray photoelectron spectroscopy (XPS) measurements. This comparison is shown in Supp, Fig. 7. Note that, despite the presence of a small amount of additional enodfullerene material above the first chemisorbed monolayer, the width of the C 1s peak for Ar@C_{60}/Ag(111) is significantly narrower than that for empty C_{60} on Ag(111). We attribute this to the difference in sample preparation conditions. Pedio *et al.* deposited fullerene molecules onto a sample that was at room temperature. The Ar@C_{60} monolayer was instead formed via deposition onto a Ag(111) crystal held at 180 K, circumventing "nanopitting" at the fullerene-Ag(111) interface[23] and leading to both a weaker and more homogeneous cage-surface interaction.

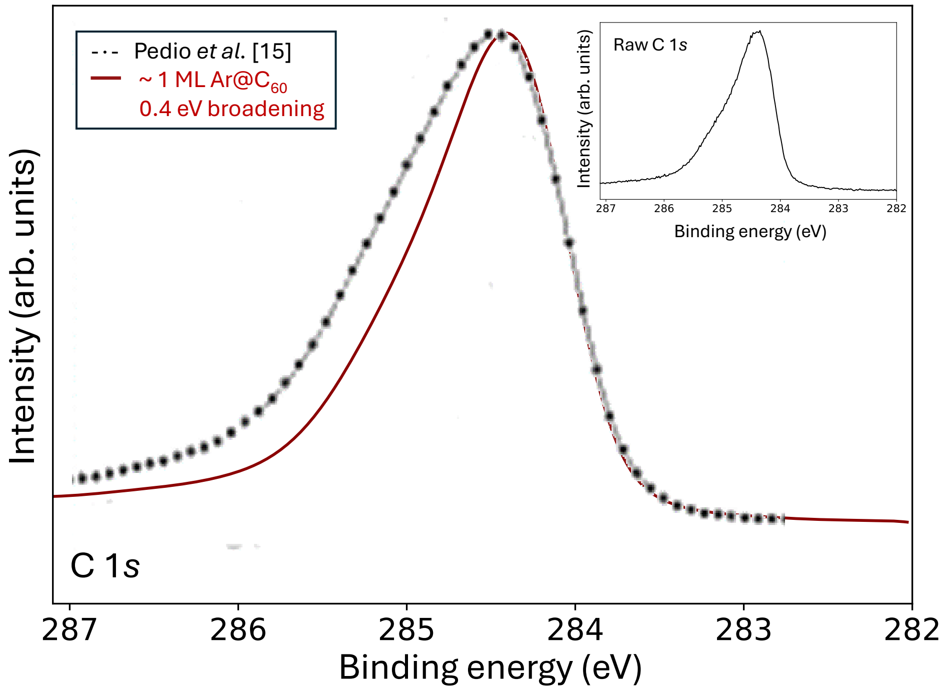

Supp. Fig. 7. Comparison of C 1s spectrum for monolayer C_{60} and Ar@C_{60} on Ag(111). Red solid line: C 1s spectrum for ∼ 1 ML Ar@C_{60} on Ag(111) artificially broadened by convolution with a Gaussian of 0.4 eV FWHM to match experimental resolution of Pedio *et al.*[20], who reported C 1s X-ray photoelectron spectroscopy (XPS) spectra for a variety of C_{60}-on-metal systems, including C_{60} on Ag(111). Pedio *et al.*'s spectrum is shown as the filled-circles-with-line curve and has been digitally cut from Fig. 4 of their paper[20], scaled appropriately, aligned, and superimposed on the broadened spectrum for Ar@C_{60}. (The original, unbroadened C 1s spectrum for ∼ 1 monolayer of Ar@C_{60} on Ag(111) is shown in the inset.) Source data are the same as those for Supp. Fig. 6.

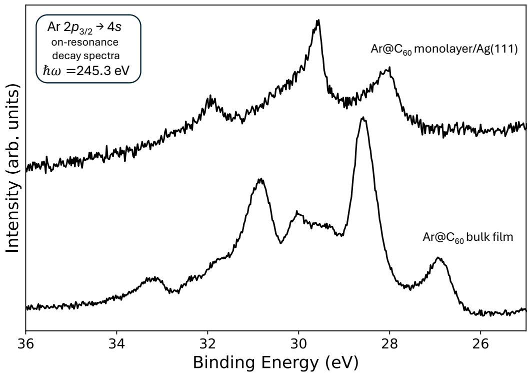

Supp. Fig. 8. Decay spectra for multilayer vs monolayer. Fig. 5 of the main paper shows a comparison between the Auger/spectator decay spectra for multilayer and monolayer coverages, following removal of a linear background and a shift of binding energy of the latter to bring it into alignment with the multilayer spectrum. Here we show the raw spectra before adjustment (other than removal of a constant background for display purposes). Source data available as Source Data files.

Supplementary Note 5. Decay spectra for multilayer and monolayer Ar@C$_{60}$ coverage

In Fig. 5 of the main paper we show a comparison of the Auger spectrum for an Ar@C$_{60}$ multilayer and monolayer for which we have aligned the corresponding Auger peaks and also subtracted a linear background from the spectrum for the Ar@C$_{60}$ monolayer. For completeness, we show here (Fig.8.) the raw data before adjustment.

Supplementary Note 6. Population analysis: Argon and carbon contributions to the 4*s* excited state

We found that the quantification of argon and carbon contributions to the excited state is less straightforward than one might have anticipated (or hoped for). Essentially, we tackle the notion of "mixing of argon and fullerene orbitals" directly by decomposing each orbital $\psi(\mathbf{r})$ into contributions from the argon atom and the carbon atoms in the fullerene cage. There are, however, many decomposition schemes, with each giving different decompositions that have varying degrees of physicality.

The most popular decomposition scheme is that of Mulliken[24], which simply computes the trace of the density matrix block corresponding to basis functions centred on the atom of interest while ignoring all off-diagonal blocks (see also Section 3.9.3 of the Multiwfn manual). Even though this scheme gives reasonable results most of the time, it is susceptible to producing negative decomposition values, which are unphysical. The Ar-contributions to the relevant molecular orbitals in the ground and excited calculations as calculated using Mulliken's decomposition scheme are shown in the Excel sheet, `popana.xlsx`, uploaded as part of the data repository.

It can be seen that, for all occupied (restricted) molecular orbitals in the ground state, and for all occupied α molecular orbitals except the distorted $4s$ one in the excited state, Mulliken's Ar-contributions are sensible. However, for the unoccupied $4s$ molecular orbital in the ground state and the occupied distorted $4s$ in the excited state, Mulliken's Ar-contributions are strongly negative, which means we cannot use this scheme to say anything meaningful about the molecular orbitals of interest to us.

We must therefore seek a more sensible alternative decomposition scheme. The main issue that these schemes must deal with is the balanced treatment of the cross terms that are ignored in Mulliken's scheme. One attempt is that by Stout and Politzer[25] (see also Section 3.9.6 of the Multiwfn manual), but this turns out to be even worse than Mulliken's, as seen in the even more negative Ar-contributions in both the ground and excited $4s$ molecular orbitals.

Other attempts include the use of Becke's weighting function[26] (see also Section 3.9.8 of the Multiwfn manual) and Hirshfeld's atomic charge[27] (see also Section 3.9.1 of the Multiwfn manual). However, both of these methods rely on a numerical real-space integration scheme for the electron density and give very small Ar-contributions for both the ground and excited $4s$ molecular orbitals, which is clearly inconsistent with the fact that the coefficients for these molecular orbitals contain relatively much larger values for the basis functions centred on the Ar atom compared to those for the basis functions centred on the C atoms of the cage.

Fortunately, there exists an alternative by Ros and Schuit[28], *C-squared population analysis (SCPA)*, that makes use of the coefficients directly (rather than via numerical real-space integrations) and that also ensures that the atom contributions are never negative (see also Section 3.9.5 of the Multiwfn manual). The Ar-contributions to the ground and excited $4s$ molecular orbitals in the SCPA scheme now appear sensible. The ground unoccupied $4s$ molecular orbital has $\sim 92.17\%$ contribution from the argon atom, which means that there is only $\sim 7.83\%$ of mixing with all the carbon atoms on the cage. On the other hand, the excited occupied and distorted $4s$ molecular orbital only has $\sim$13.38 % contribution from the argon atom, indicating that there is now significant mixing with the cage.

Supplementary Note 7. Orbital symmetry considerations

In the main paper, we note that we used QSYM^2[29] to extract the symmetry components of the excited state. The following approach was adopted when assessing the symmetry decompositions.

By applying the projection operator

$$\hat{\mathcal{P}}^{\Gamma} = \frac{d_\Gamma}{|\mathcal{G}|} \sum_{\hat{g} \in \mathcal{G}} \chi^{\Gamma}(\hat{g})^* \hat{g} \tag{1}$$

to a molecular orbital ψ, where $\mathcal{G}$ is the symmetry group of the system, Γ an irreducible representation of $\mathcal{G}$ with dimensionality d_Γ, and $\chi^\Gamma(\hat{g})$ the character of the symmetry operation $\hat{g}$ in Γ, we can determine the Γ-composition of ψ:

$$c_\Gamma(\psi) = \langle\psi|\hat{\mathcal{P}}^\Gamma|\psi\rangle = \frac{d_\Gamma}{|\mathcal{G}|}\sum_{\hat{g}\in\mathcal{G}} \chi^\Gamma(\hat{g})^* \langle\psi|\hat{g}|\psi\rangle , \tag{2}$$

where it can be shown that

$$\sum_\Gamma c_\Gamma(\psi) = \langle\psi|\psi\rangle = 1 \tag{3}$$

if ψ is normalised. This procedure can be carried out using QSYM^2[29]. Since the calculations for Ar@C_{60} were done with a perfect icosahedral structure, the group $\mathcal{G}$ is the icosahedral group $\mathcal{I}_h$ and the possible irreducible representations are A_g, T_{1g}, T_{2g}, F_g, H_g, A_u, T_{1u}, T_{2u}, F_u, and H_u. The above procedure applied to the ground- and excited-state $4s$ molecular orbitals yields the decomposition shown in tab:decom.

We know from the $O(3) \supset \mathcal{I}_h$ subduction that the first few even spherical harmonics have the following symmetries in $\mathcal{I}_h$:

$$\begin{aligned} &S\ \ (l=0) \to A_g, \\ &D\ \ (l=2) \to H_g, \\ &G\ \ (l=4) \to F_g \oplus H_g, \\ &I\ \ \ (l=6) \to A_g \oplus T_{1g} \oplus F_g \oplus H_g. \end{aligned}$$

Hence, with the decompositions shown above, we deduce that the ground-state $4s$ molecular orbital consists approximately of $\sim$ % S-component, whereas the excited-state distorted $4s$ molecular orbital consists approximately of $\sim$ 76% S-symmetry component, $\sim$ 23% D-symmetry component, and less than 1% G-symmetry component. A direct decomposition of each molecular orbital into spherical-harmonic components is possible but is beyond the scope of this work.

Supp. Table 1. Irreducible representation decompositions for the ground- and excited-state $4s$ molecular orbitals in the $\mathcal{I}_h$ structure. Both of these molecular orbitals are even with respect to spatial inversion, and so only the *gerade* irreducible representations need to be considered.

$\mathcal{I}_h$	ground-state $4s$ (%)	excited-state $4s$ (%)
A_g	100.00	76.06
T_{1g}	0.00	0.00
T_{2g}	0.00	0.00
F_g	0.00	0.67
H_g	0.00	23.27

Supplementary Note 8. The use of ΔSCF via the maximum overlap method (MOM); comparison with time-dependent DFT calculations

We used ΔSCF with high confidence, as numerous studies have showed that ΔSCF can predict excitation energies with accuracy comparable to, and occasionally surpassing, that of time-dependent DFT (TD-DFT) when employing the maximum overlap method (MOM) to optimise the excited states[30–32]. In Table 2., we compare the calculated excitation energies for the Ar $2p \to 4s$ transition between MOM and TD-DFT in two different exchange–correlation functionals. It can be seen that while ΔSCF/MOM somewhat overestimates the excitation energies, TD-DFT *severely* underestimates them.

We attribute the discrepancy between the accuracies of ΔSCF/MOM and TD-DFT compared to experimental results to the fact that the orbitals in TD-DFT calculations are unrelaxed. As such, any TD-DFT excited states involving the unrelaxed unoccupied $4s$ orbital of the ground-state DFT calculation would not be able to pick up the qualitative distortion due to the mixing with the C_{60} cage in the excited state as demonstrated by ΔSCF via the MOM. Moreover, using ΔSCF via the MOM allows us to visualise the orbitals involved in the transition, therefore gaining a clearer qualitative understanding of the targeted excited states, which would not be possible with TD-DFT.

Supp. Table 2. Calculated excitation energies for the Ar $2p \to 4s$ transition. All energy values are in eV and correspond to singlet excited states with $M_S = 0$ and $\langle \hat{S}^2 \rangle \approx 0$. This was ensured in ΔSCF via the approximate spin purification equation[33, 34] $E_{\langle \hat{S}^2 \rangle \approx 0} \approx 2E_{\langle \hat{S}^2 \rangle \approx 1} - E_{\langle \hat{S}^2 \rangle \approx 2}$, where the MOM was used to converge into Kohn–Sham states with $M_S = 0$ and $\langle \hat{S}^2 \rangle \approx 1$ and $\langle \hat{S}^2 \rangle \approx 2$ that correspond to the Ar $2p \to 4s$ transition. The experimentally measured energy for the Ar $2p_{3/2} \to 4s$ transition is 245.3eV (Figures 2 and S1).

	ΔSCF/MOM	TDDFT
PBE/6-31++G**	248.91	230.63
B3LYP/6-31++G**	250.07	236.93

Quantitative evaluation of delocalisation time represents a significant challenge requiring the use of electron quantum dynamics methods such as the wave-packet propagation (WPP) approach[35, 36]. An important requirement for WPP methods is a high-level quantitative description of the excited state wavefunction, which plays a role of the initial condition for the Cauchy problem. This remains a largely unsolved, non-trivial task for DFT methods[35], which becomes even more complicated for larger

chemical systems such as Ar@C_{60} and Ar@C_{60}/Ag(111). Although MOM-DFT might, in principle, be a good source of the initial wavefunction required for the WPP calculations, implementing a theoretical framework to solve this long-standing problem goes far beyond the scope of the present paper.

Supplementary Note 9. Relativistic considerations

Although a relativistic treatment would further enhance the theoretical calculations, we found that it was difficult to include relativistic effects via TD-DFT in such a way that enables us to elucidate and distinguish the two transitions Ar $2p_{3/2} \rightarrow 4s$ and Ar $2p_{1/2} \rightarrow 4s$. For example, spin–orbit coupling calculations in contemporary quantum chemistry packages such as ORCA tend to give coupling matrix elements between multiplets that arise from a non-relativistic TD-DFT calculation, and while this might be able to incorporate some relativistic effects into the computed excitation energies, we found that it was not straight-forward at all to obtain physical insights into the spin–orbit-coupled excitation energies that would give us the understanding or insights we sought.

Instead, we performed Dirac–Hartree–Fock (DHF) calculations[37, 38] including Breit and Gaunt interactions[39, 40] in a j-adapted Gaussian basis set as implemented in PySCF[41]. As a single-determinantal method, DHF provides access to spin–orbit-coupled four-component molecular orbitals (bispinors) which allows us to identify the Ar $2p_{3/2}$ and $2p_{1/2}$ bispinors in the ground state of Ar@C60 easily. In fact, the DHF/6-31++G** calculation for the ground state yields doubly degenerate Ar $2p_{1/2}$ bispinors and quadruply degenerate Ar $2p_{3/2}$ bispinors that are separated by 2.13 eV, in good agreement with experimental results (Figure 2.).

Supplementary Note 10. The Z+1 approximation: ground state K@C_{60}

Key Kohn-Sham molecular orbitals for K@C_{60} calculated at the PBE/ 6-31++G** level of theory are shown in Fig. 9.. As noted in the main paper, while there is indeed some similarity between the Ar $2p \rightarrow 4s$ excited-state Ar@C_{60} system and the ground state K@C_{60} system by virtue of the $Z + 1$ approximation, for the latter there exists both a first-order electron transfer from K to C_{60} to generate the ion pair $K^+(C_{60})^-$ and a second-order back-donation from $(C_{60})^-$ to K^+ to reduce the charge separation in accordance with Pauling's principle of electroneutrality. On the other hand, in excited-state Ar@C_{60} there exists only a partial charge transfer from Ar to C_{60} via the distortion of the occupied Ar $4s$ molecular orbital induced by the core hole, without any back-donation from the cage.

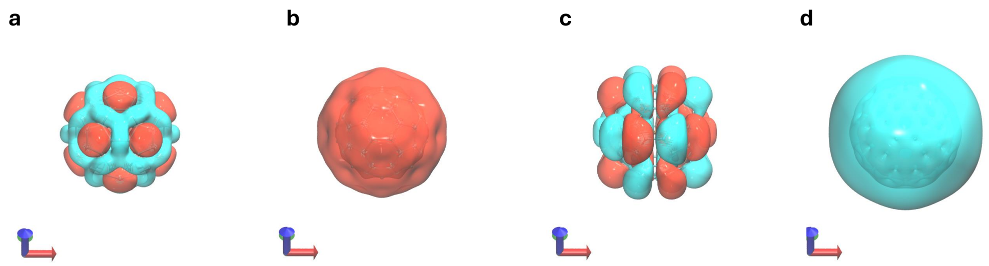

Supp. Fig. 9. Kohn-Sham molecular orbitals for K@C_{60}, calculated at the PBE/6-31++G level of theory.** All K contribution values were determined using the SCPA decomposition scheme discussed in Section 6 above. **(a)** Occupied, -15.08 eV, A_g, 53% K; **(b)** occupied, -12.27 eV, A_g,60% K; **(c)** HOMO, -4.27 eV, T_{1u}, 2% K; **(d)** unoccupied, -1.45 eV, A_g, 98% K. Atomic coordinates for DFT calculations provided as Supplementary Data 9.

Supplementary Note 11. Determining the Ar-Ag(111) separation: Supplementary density functional theory methods

In addition to the LDA PAW-DFT results described in the main paper, we have carried out an extensive set of DFT calculations at various levels of theory in order to predict the separation of the encapsulated argon atom from the Ag(111) surface for the Ar@C_{60} monolayer sample. The results are summarised in Table 3. Remarkably, we find best agreement with the experimentally measured (i.e. NIXSW derived) value of the Ar-Ag(111) separation from the most modest level of theory, i.e. LDA. Despite our expectation that good agreement would require careful implementation of dispersion force corrections, this surprisingly turned out to give substantially poorer agreement with experiment (as is clear from Table 3). A set of images of the various molecular adsorption configurations is available at the data repository for this paper.

	**bond-site**	**hex-hollow**	**hex-top**	**66 bond-hollow**	**66 bond-top**
**LDA**	5.701	5.759	5.605	5.685	5.549
**PBE-D2**	5.822	5.831	5.996	5.777	5.718
**PBE-D3**	5.847	5.839	5.770	5.820	5.740
**SCAN**	-	-	-	-	5.736
**SCAN-D3**	-	-	-	-	5.694

Supp. Table 3. The Ar-Ag separations (in Å) calculated using different DFT functionals. The labels of the columns use the nomenclature molecular orientation-binding site. The experimentally determined value (see Fig. 3 of the main paper and associated discussion) is 5.54 ± 0.04 Å.

Supplementary references

[1] Morscher, M., Seitsonen, A. P., Ito, S., Takagi, H., Dragoe, N., and Greber, T., Strong $3p - T_{1u}$ hybridization in Ar@C_{60}, *Phys. Rev. A.* **82**, 051201(R) (2010)

[2] Wurth, W., Rocker, G., Feulner, P., Scheuerer, R., Zhu, L., and Menzel, D. Core excitation and deexcitation in argon multilayers: Surface- and bulk-specific transitions and autoionization versus Auger decay, *Phys. Rev. B* **47** 6697 (1993)

[3] Måtensson, N., Karis, O., and Nilsson, A. Resonant processes in the soft X-ray regime, *J.Elec. Spec. Rel. Phen.* **100**, 379 (1999)

[4] Sandell, A., Brühwiler, P.A., Nilsson, A, Bennich, P, Rudolf, P., and Mårtensson, N. Nature of the ns-derived states for an isolated alkali atom on a surface, *Surf. Sci.* **429** 309 (1999)

[5] Sandell, A., Hjortstam, O., Nilsson, A., Brühwiler, P. A., Eriksson, O., Bennich, P., Rudolf, P., Wills, J. M., Johansson, B., and Mårtensson, N. Bonding of an isolated K atom to a surface: Experiment and theory, *Phys. Rev. Lett.* **78** 4994 (1997)

[6] Lizzit, S., Larciprete, R., Lacovig, P., Kostov, K. L., and Menzel, D. Ultrafast charge transfer at monolayer graphene surfaces with varied substrate coupling, *ACS Nano* **7**, 4359 (2013)

[7] Lizzit, S., Zampieri, G., Kostov, K. L., Tyuliev, G., Larciprete, R., Petaccia, L., Naydenov, B., and Menzel, D., Charge transfer from core-excited argon adsorbed on clean and hydrogenated Si(100): ultrashort timescales and energetic structure, *New. J. Phys.* **11**, 053005 (2009)

[8] Zhao, J., Feng M., and Petek, H. The superatom states of fullerenes and their hybridization into the nearly free electron bands of fullerenes. *ACSNano* **3**, 853 (2009)

[9] Wurth, W., Feulner, P., and Menzel, D., Resonant excitation and decay of adsorbate core holes, *Physica Scripta* **T41**, 213 (1992)

[10] Johansson, F. O. L. *et al.*, Tailoring ultra-fast charge transfer in MoS_2, *Phys. Chem. Chem. Phys.* **22**, 10335 (2020)

[11] Lizzit, S., Zampieri, G., Kostov, K. L., Tyuliev, G., Larciprete, R., Petaccia, L., Naydenov, B., and Menzel, D., Charge transfer from core-excited argon adsorbed on clean and hydrogenated Si(100): ultrashort timescales and energetic structure, *New. J. Phys.* **11**, 053005 (2009)

[12] Lizzit, S., Larciprete, R., Lacovig, P., Kostov, K. L., and Menzel, D. Ultrafast charge transfer at monolayer graphene surfaces with varied substrate coupling,

ACS Nano **7**, 4359 (2013)

[13] Wurth, W. and Menzel, D., Ultrafast electron dynamics at surfaces probed by resonant Auger spectroscopy, *Chem. Phys.***251**, 141 (2000)

[14] Mårtensson, N. and Nilsson, A., Autoionization as a tool for studying adsorbed atoms and molecules, *J. Elec. Spec. Rel. Phen.* **72**,1 (1995)

[15] Karis, O., Nilsson, A., Weinelt, M., Wiell, T., Puglia, C., Wassdahl, N., Mårtensson, N., Samant, M., and Stöhr, J., One-step and two-step description of deexcitation processes in weakly interacting systems, *Phys. Rev. Lett.* **76**, 1380 (1996)

[16] Bommel, S., Kleppmann, N., Weber, C., Spranger, H., Schäfer, P., Novak, J., Roth, S. V., Schreiber, F., Klapp, S. H. L., and Kowarik, S. Unravelling the multilayer growth of the fullerene C_{60} in real time, *Nature Comms.* **5** 5388 (2014)

[17] Mayer, J., Khairy, K., and Howard, J. Drawing an elephant with four complex parameters, *Am. J. Phys.* **78**, 648 (2010)

[18] Doniach S. and Sunjic M., Many-electron singularity in X-ray photoemission and X-ray line spectra from metals, *J. Phys. C* **3**, 285 (1970)

[19] Biswas, C., Shukla, A.K., Banik, S., Barman, S. R., Chakrabarti, A. Argon nanobubbles in Al(111): A photoemission study, *Phys. Rev. Lett.* **92** 115506-1 (2004)

[20] Pedio M., Hevesi, K., Zema, N., Capozi, M., Perfetti, P., Gouttebaron, R., Pireaux, J. -J., Caudano, R., and Rudolf, P., C_{60}/metal surfaces: adsorption and decomposition, *Surf. Sci.* **437**, 249 (1999)

[21] Gibson, A. J., Temperton, R. H., Handrup, K., and O' Shea, J.N. Resonant core spectroscopies of the charge transfer interactions between C_{60} and the surfaces of Au(111), Ag(111), Cu(111) and Pt(111). *Surf. Sci.* **657**, 69 (2017)

[22] In a somewhat related vein, Meier *et al.* (*Nature Comm* **6** 8112 (2015)) have observed a similarly surprising dependence of the bulk dielectric constant of $H_2O@C_{60}$ on the nuclear spin (ortho vs para) of the encapsulated water molecule.

[23] Li, H. I, Pussi, K., Hanna, K. J., Wang, L-. L., Johnson, D. D., Cheng, H. -P., Shin, H., Curtarolo, S., Moritz, W., Smerdon, J. A., McGrath, R., and Diehl, R.D., Surface geometry of C_{60} on Ag(111), *Phys. Rev. Lett.* **103**, 056101 (2009)

[24] Mulliken, R. S. Electronic population analysis on LCAO-MO molecular wave functions. I. *J. Chem. Phys.* **23**, 1833 (1955)

[25] Stout, E. W. and Politzer, P. An investigation of definitions of the charge on an atom in a molecule, *Theor. Chim. Acta* **12**, 379 (1968)

[26] Becke, A. D. A multicnter numerical integration scheme for polyatomic molecules, *J. Chem. Phys.* **88**, 2547 (1988)

[27] Hirshfeld, F. L. Bonded-atom fragments for describing molecular charge densities, *Theor. Chim. Acta* **44**, 129 (1977)

[28] Ros, P. and Schuit, G. C. A., Molecular orbital calculations on copper chloride complexes, *Theor. Chim. Acta.* **4**, 1 (1966)

[29] Huynh, B. C., Wibowo-Teale, M, and Wibowo-Teale, A. M. QSYM^2: A quantum symbolic symmetry analysis program for electronic structure, *J. Chem. Theor. Comp.* **20**, 114 (2024)

[30] Bourne Worster, S., Feighan, O, and Manby, F. R. Reliable transition properties from excited-state mean-field calculations, *J. Chem. Phys.***154**, 124106 (2021)

[31] Bogo, N. and Stein, C. J. Benchmarking DFT-based excited state methods for intermolecular charge-transfer excitations, *Phys. Chem. Chem. Phys.* **26**, 21575 (2024)

[32] Kowalczyk, T., Yost S. R., and Voorhis, T. V. Assessment of the ΔSCF density functional theory approach for electronic excitations in organic dyes, *J. Chem. Phys.* **134**, 054128 (2011)

[33] Daul, C. Density functional theory applied to the excited states of coordination compounds, *Int. J. Quantum Chem.* **52**, 867 (1994)

[34] Ziegler, T, Rauk, A., and Baerends, E. J. On the calculation of multiplet energies by the Hartree-Fock-Slater method. *Theor. Chim. Acta* **43**, 261 (1977)

[35] Aguilar-Galindo, F., Borisov, A. G., and Dìaz-Tendero, S., Ultrafast dynamics of electronic resonances in molecules adsorbed on metal surfaces: A wave packet propagation approach, *J. Chem. Theor. Comp.* **17**, 639 (2021)

[36] Gauyacq, J. P. and Borisov, A. N., Excited electron transfer between a core-excited $\mathrm{Ar}^*(2p_{3/2}^{-1}4s)$ atom and the metal substrate in the Ar/Cu(111) system, *Phys. Rev. B* **69**, 235408 (2004)

[37] Mohanty, A. and Clementi, E. Dirac-Fock self-consistent field method for closed-shell molecules with kinetic balance and finite nuclear size, *Int. J. Quantum Chem.* **39**, 487 (1991)

[38] Sun, S., *et al.*, Efficient four-component Dirac-Coulomb-Gaunt Hartree-Fock in the Pauli spinor representation. *J. Chem. Theory Comput.* **17**, 3388 (2021)

[39] Briet, G. The effect of retardation on the interaction of two electrons. *Phys. Rev.* **34**, 553 (1929)

[40] Malli, G. L. Dirac-Fock-Briet-Gaunt calculations for tungsten hexacarbonyl $W(CO)_6$. *J. Chem. Phys.* **144**, 194301 (2016)

[41] Sun, Q., *et al.*, Recent developments in the PySCF program package. *J. Chem. Phys.* **153**, 024109 (2020)
