# Supplementary material for: Timing the escape of a photoexcited electron from a molecular cage
Source: Nat Commun. 2025 May 31;16:5062. doi: 10.1038/s41467-025-60260-z (PMC12126515; doi:10.1038/s41467-025-60260-z)
Supplement: Supplementary file 2 — Description of Additional Supplementary Files [file 41467_2025_60260_MOESM2_ESM.pdf]

Description of Additional Supplementary Files:

Supplementary Data 1: Coordinate file related to DFT calculations of Fig. 4

Supplementary Data 2: Coordinate file related to DFT calculations of Fig. 3

Supplementary Data 3: Coordinate file related to DFT calculations of Supp. Fig. 9
